# Are Trace Element Concentrations in Lung Cancer Tissue Associated with Metastasis?

**DOI:** 10.5152/eurasianjmed.2021.20407

**Published:** 2021-10

**Authors:** Omer Araz, Aslı Araz

**Affiliations:** 1 Department of Pulmonary Diseases, Atatürk University School of Medicine, Erzurum, Turkey; 2 Department of Physics, Atatürk University School of Sciences, Erzurum, Turkey

**Keywords:** Lung cancer, Metastasis, Lead, Aluminum

## Abstract

**Objective:**

Lung cancer is among the leading causes of cancer-related deaths. Many exogenous and endogenous factors are associated with the development and progression of this cancer. Among these factors are trace elements, which have many biological functions despite their low concentrations in the body and may disrupt cellular functions and contribute to tumorigenesis when present in excessive or insufficient quantities. In this study, we performed elemental analysis of lung cancer tissues to evaluate the role of trace element concentrations in the formation of metastasis in lung cancer.

**Materials and Methods:**

Lung cancer tissue specimens were collected from 65 patients with different cancer stages and histological subtypes for elemental analysis. After the tissues were embedded in paraffin blocks and prepared, the concentrations of 19 elements were analyzed by using inductively coupled plasma-optical emission spectroscopy (ICP-OES). All of the patients included in the study underwent diagnosis, treatment, and follow-up in our center between 2015 and 2020.

**Results:**

Comparison of trace element concentrations in three different lung cancer subtypes according to cancer stage showed that lead (Pb) and aluminum (Al) concentrations increased significantly as cancer stage advanced (*P* < .0001 for both). Trace element concentrations did not differ significantly based on patient sex or age.

**Conclusion:**

Lead and aluminum concentrations in the tissues of lung cancer patients may contribute to the formation of metastases, which have a major impact on the prognosis of lung cancer.

## Introduction

Lung cancer is the leading cause of cancer-related deaths worldwide and ranks as the first and third most common cancer in men and women, respectively. Mortality rates remain high in lung cancer despite advances in surgical and medical treatment methods. Surgical treatment is the main treatment option for patients with operable stage 1 and 2 lung cancers, while chemotherapy and radiotherapy are used in patients with stage 3 and 4 lung cancer.^1^

Metastatic status is a major determinant of treatment approach and prognosis. There is rich literature data on the numerous factors and pathways that play a role in the initiation of metastasis, such as tumor suppressor genes, proliferative activity markers, and the epithelial-to-mesenchymal transition.^2,3^ Although much has been elucidated regarding the cause of metastasis, there are still many gaps in our knowledge. One of these areas is trace elements.^4^

The aim of the present study was to evaluate whether trace elements, which have important biological functions despite their low concentrations in body tissues, have a role in lung cancer metastasis.

## Materials and Methods

Tissues from 65 patients with newly diagnosed lung cancer of various types and stages were examined using trace element analysis. Patients with a history of occupational exposure to any specific substance and those with metabolic or kidney diseases that may lead to element accumulation were excluded (n = 17). All patients underwent diagnosis, treatment, and follow-up in our center between 2015 and 2020.

### Preparation of tissue samples

Lung cancer tissue specimens collected from patients with different histological subtypes and stages during bronchoscopy, endobronchial ultrasound (EBUS), or surgery were obtained from the pathology department of the Atatürk University School of Medicine. The paraffin-embedded tissue samples were dried in the incubator at 80 °C for 24 hours. The dried tissue samples weighed approximately 0.5 g on a precision scale. Microwave-safe containers were cleaned and prepared for measurement by placing them in the microwave oven with 5 mL HNO_3_. The dried tissue samples were placed in the pressurized containers, followed by 3 mL of 30% H_2_O_2_ and 2 mL of 65% HNO_3_.

After solubilizing the samples in the microwave oven, the containers were cooled at room temperature. The cooled solutions were filtered (Whatman filter papers, grade 42, 125 mm diameter) and transferred to 25 mL volumetric flasks. Distilled/deionized water was added to the flasks to bring the final volume to 25 mL, which was aliquoted into a pair of 14 mL tubes for analysis.

Trace element analysis of the solubilized tissue samples was performed by using inductively coupled plasma-optical emission spectroscopy (ICP-OES) (Perkin-Elmer, Optima 2100 DV, ICP/OES, Shelton, CT).

### The ICP-OES instrument

The working principle of the ICP-OES analyzer is that a solubilized sample is vaporized by high-temperature plasma to excite the atomized elements in the sample, which then release emission rays that are measured by an appropriate detector to determine the concentration of elements in the sample. ICP-OES provides advantages such as enabling analysis of low concentrations, measuring with high accuracy, precision, and sensitivity, and being simple to perform.^5^

### Analyzed elements

A total of 19 elements were detected in the ICP-OES analysis of the lung cancer tissue samples: calcium (Ca), magnesium (Mg), sodium (Na), potassium (K), iron (Fe), copper (Cu), zinc (Zn), manganese (Mn), aluminum (Al), boron (B), barium (Ba), cadmium (Cd), chromium (Cr), molybdenum (Mo), nickel (Ni), phosphorus (P), lead (Pb), sulfur (S), and selenium (Se).

### Cancer staging

The patients were staged according to the eighth edition of the classification of malignant tumors system.^6^ The staging was done via bronchoscopy, EBUS, positron-emitting tomography/computed tomography (PET-CT), brain MRI examination, and surgery.

### Statistical analysis

The data were analyzed using SPSS version 18 statistical software (SPSS Inc.; Chicago, IL, USA). Mann-Whitney *U* test was used to compare element concentrations in lung cancer tissues according to stage and histological subtype. Relationships between element concentrations and patient age and sex were evaluated using Pearson correlation analysis. A *P* value of less than .05 was considered statistically significant.

## Results

A total of 65 patients were included. Their demographic and clinical data are summarized in Table 1. Three different histopathological subtypes were identified in the patients: squamous cell carcinoma (n = 26), adenocarcinoma (n = 24), and small cell lung cancer (n = 15).

Sex-based comparisons revealed no significant differences in element concentrations between men and women (*P* > .05). There were also no significant differences in element concentrations based on age (*P* > .05). Statistically significant relationships were observed between all histological subtypes of lung cancer and concentrations of lead and aluminum (*P* < .05).

**Figure 1.**
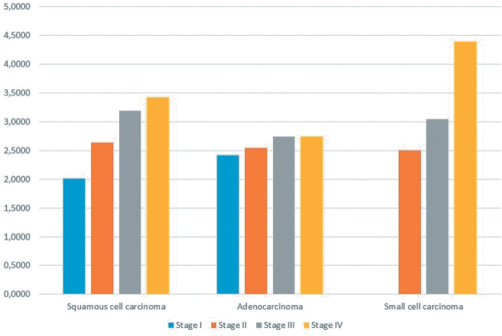
Mean lead (Pb) concentrations detected in the different histological subtypes of lung cancer according to stage

**Figure 2.**
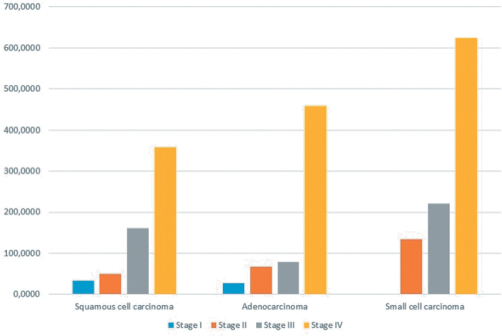
Mean aluminum (Al) concentrations detected in the different histological subtypes of lung cancer according to stage

Evaluation of the relationships between trace elements and cancer stage showed that tissue concentrations of lead and aluminum increased significantly with higher lung cancer stages, regardless of histological cell type (*P* < .0001 for both) (Figures 1 and 2).

## Discussion

Metastasis is an important determinant of prognosis in lung cancer. This study investigated whether trace elements play a role in the development of metastasis, and the results demonstrated that lead and aluminum were positively correlated with lung cancer stage, suggesting that these elements may be an important contributing factor in lung cancer progression to advanced stages, especially metastasis.

Elements are known to have a role in many diseases, particularly cancers, by disrupting antioxidant defense and immune responses. Trace elements act as enzyme components in biological systems or as catalysts in intracellular chemical reactions. Therefore, deficient or excessive levels of numerous elements are known to promote the development of many diseases, including various types of cancer. The main elements shown to cause cancer include beryllium, chromium, cobalt, nickel, arsenic, cadmium, antimony, lead, silver, and platinum, while the impact of manganese, iron, copper, zinc, selenium, and strontium on cancer development has not been clearly demonstrated.^4,7,8^

In our study, the high level of lead detected in lung cancer tissue, especially from patients with advanced (stage 3/4) cancer, may be related to the adverse effects of lead on macrophages, a component of innate immunity found throughout the body. Lead exposure substantially reduces the protective activity of macrophages against various pathogens, as well as skewing the immune system toward Th2 responses. More relevant to the present study, lead also causes the generation of reactive oxygen and nitrogen species, resulting in lipid peroxidation and DNA damage, which is known to contribute to cancer formation.^9,10^ With these properties, we believe that high levels of Pb in the tissues of patients with advanced lung cancer, together with many other pathways and factors, may have impaired immune function and antitumor defenses, thereby promoting the formation of metastases.

In the literature, aluminum has been associated with many health problems, especially respiratory diseases, malignancies such as bladder, lymphatic, and hematologic cancers, and conditions such as aplastic anemia.^11,12^ In a recent study conducted in a human colorectal cancer cell line, aluminum was shown to have wide-ranging effects on cells, such as promoting migration and invasion and suppressing adhesion. Exposure to aluminum also altered the expression and secretion of various proteins associated with angiogenesis, tumorigenesis, and metastasis, including E-cadherin, vimentin, Snail, transforming growth factor-β, matrix metalloproteinases 7 and 9, and vascular endothelial growth factor.^13^ That study demonstrated that gastrointestinal tract exposure to aluminum may be a risk factor for the initiation of metastasis in colorectal cancer cells. We believe that this element may affect the same systems and immunological signaling pathways to promote metastasis in lung cancer.

One of the limitations of our study is the small sample size. Multicenter studies are needed to corroborate our findings. However, this study is the first in the literature to investigate the relationship between lung cancer stage/metastasis and trace element levels measured using the highly sensitive method of ICP-OES.

As in many other malignancies, trace elements play a role in the development and course of lung cancer. The findings of our study suggest that lead and aluminum may also influence disease progression and metastasis in addition to other factors. Studies with larger patient series may support our results and better demonstrate the role of these trace elements in lung cancer metastasis.

**Main Points**

- Trace elements play a role in the development and course of lung cancer.
- Lead and aluminum concentrations in the tissues of lung cancer patients may contribute to the formation of metastases
- Lead and aluminum are more associated in small cell lung cancer metastases

## Figures and Tables

**Table 1.** Demographic and Clinical Characteristics of the Lung Cancer Patients

	Squamous Cell Carcinoma	Adenocarcinoma	Small Cell Carcinoma
Number of cases	26	24	15
Age (year)	60 ± 8.2	57.7 ± 7.4	60.5 ± 9.8
Gender (female/male)	(3/23)	(7/17)	(4/11)
Cigarette use (pack-years)	49.5 ± 18.7	43.4 ± 17.8	38.8 ± 20.6
Stage I	3	9	-
Stage II	8	4	3
Stage III	8	4	6
Stage IV	7	7	6

## References

[1] Siegel RL Miller KD Jemal A. Cancer statistics, 2020. CA Cancer J Clin. 2020;70(1):7. 10.3322/caac.21590 31912902

[2] Gavert N Ben-Ze’ev A. Epithelial–mesenchymal transition and the invasive potential of tumors. TrendsMol Med. 2008;14(5):199-209. 10.1016/j.molmed.2008.03.004 18406208

[3] Araz O Demirci E Ucar EY, . Roles of Ki-67, p53, transforming growth factor-β and lysyl oxidase in the metastasis of lung cancer. Respirology. 2014;19(7):1034-1039. 10.1111/resp.12345 24995672

[4] Araz Ö Araz A Yılmazel Uçar E Demirci E Aydın Y Akgün M. The effect of surgical specimen-derived phosphorus and lead concentrations in non small cell lung cancer patients on disease course. Tuberk Toraks. 2018;66(4):334-339. 10.5578/tt.67834 30683029

[5] Carpenter R. The analysis of some evidential materials by inductively coupled plasma-optical emission spectrometry. Forensic Science International. 1985;27(3):157-163. 10.1016/0379-0738(85)90152-5 3988196

[6] Goldstraw P Chansky K Crowley J, . The IASLC lung cancer staging project: Proposals for revision of the TNM stage groupings in the forthcoming (Eighth) Edition of the TNM classification for lung cancer. J Thorac Oncol. 2016;11(1):39-51. 10.1016/j.jtho.2015.09.009 26762738

[7] Patriarca M Menditto A Di Felice G, . Recent developments in trace element analysis in the prevention, diagnosis, and treatment of diseases. Microchemical J. 1998;59(2):194-202. 10.1006/mchj.1998.1599

[8] Araz Ö. Araz A Yılmazel Uçar E, . Can tissue elemental analysis be used to differentiate sarcoidosis and tuberculous lymphadenitis? Tuberk Toraks. 2020;68(2):126-134. 10.5578/tt.69691 32755112

[9] Jomova K Valko M. Advances in metal-induced oxidative stress and human disease. Toxicology. 2011;283(2-3):65-87. 10.1016/j.tox.2011.03.001 21414382

[10] Kasten‐Jolly J Lawrence DA. Lead modulation of macrophages causes multiorgan detrimental health effects. J Biochem Mol Toxicol. 2014;28(8):355-372. 10.1002/jbt.21572 24863546

[11] McClure ES Vasudevan P DeBono N Robinson WR Marshall SW Richardson D. Cancer and noncancer mortality among aluminum smelting workers in Badin, North Carolina. Am J Ind Med. 2020;63(9):755-765. 10.1002/ajim.23150 32649003 PMC7890681

[12] Zuo Y Lu X Wang X, . High-dose aluminum exposure further alerts immune phenotype in aplastic anemia patients. Biol Trace Elem Res. 2021;199(5):1743-1753. 10.1007/s12011-020-02313-6. 32761514 PMC7990755

[13] Jeong CH Kwon HC Cheng WN Kim DH Choi Y Han SG. Aluminum exposure promotes the metastatic proclivity of human colorectal cancer cells through matrix metalloproteinases and the TGF-β/Smad signaling pathway. Food Chem Toxicol. 2020;141:111402. 10.1016/j.fct.2020.111402 32437896

